# Impact of follow ups, time interval and study duration in diffusion & myelin MRI clinical study in MS

**DOI:** 10.1016/j.nicl.2023.103529

**Published:** 2023-10-12

**Authors:** Manon Edde, Francis Houde, Guillaume Theaud, Matthieu Dumont, Guillaume Gilbert, Jean-Christophe Houde, Loïka Maltais, Antoine Théberge, Moussa Doumbia, Ann-Marie Beaudoin, Emmanuelle Lapointe, Muhamed Barakovic, Stefano Magon, Maxime Descoteaux

**Affiliations:** a Imeka Solutions, Inc., Sherbrooke, QC, Canada; b Université de Sherbrooke, Sherbrooke, QC, Canada; c MR Clinical Science, Philips Healthcare Canada, Mississauga, Ontario, Canada; d Videos & Images Theory and Analytics Laboratory (VITAL), Université de Sherbrooke, Sherbrooke, QC, Canada; e Université de Sherbrooke, CIUSSS de l’Estrie-CHUS Fleurimont, Sherbrooke, QC, Canada; f Roche Pharma Research and Early Development, Neuroscience and Rare Diseases, Roche Innovation Center Basel Switzerland, F. Hoffmann-La Roche Ltd., Basel, Switzerland

**Keywords:** Diffusion MRI, Inhomogeneous magnetization transfer, Follow-ups, Time-interval, Study duration, Longitudinal study, Multiple Sclerosis, Healthy controls

## Abstract

It is currently unknown how quantitative diffusion and myelin MRI designs affect the results of a longitudinal study. We used two independent datasets containing 6 monthly MRI measurements from 20 healthy controls and 20 relapsing-remitting multiple sclerosis (RR-MS) patients. Six designs were tested, including 3 MRI acquisitions, either over 6 months or over a shorter study duration, with balanced (same interval) or unbalanced (different interval) time intervals between MRI acquisitions. First, we show that in RR-MS patients, the brain changes over time obtained with 3 MRI acquisitions were similar to those observed with 5 MRI acquisitions and that designs with an unbalanced time interval showed the highest similarity, regardless of study duration. No significant brain changes were found in the healthy controls over the same periods. Second, the study duration affects the sample size in the RR-MS dataset; a longer study requires more subjects and vice versa. Third, the number of follow-up acquisitions and study duration affect the sensitivity and specificity of the associations with clinical parameters, and these depend on the white matter bundle and MRI measure considered. Together, this suggests that the optimal design depends on the assumption of the dynamics of change in the target population and the accuracy required to capture these dynamics. Thus, this work provides a better understanding of key factors to consider in a longitudinal study and provides clues for better strategies in clinical trial design.

## Highlights

• Changes over time from 3 MRIs are similar to those obtained from 5 MRIs.
• Some differences are seen in the RR-MS dataset, but not in the healthy dataset.
• The associations with the clinical outcomes are affected by the study design.
• The effect of the design strategy is bundle- and MRI measure-dependent.
• The optimal design will depend on the dynamics of change in the target population.

## 1 Introduction

The ever-increasing number of longitudinal neuroimaging studies must be designed to address not only questions about the dynamics of brain changes over time (Klistorner et al., 2018, Kolind et al., 2022, Schuler et al., 2019) but also ethical and practical feasibility requirements, such as available resources and funding. There are therefore trade-offs between a well-sampled, high-quality dataset, and the cost of data collection in terms of time and money. In neuroimaging, the planning of MRI acquisitions is a key point based, among others, on 3 factors: the number of follow-up MRI acquisitions, the interval between MRI acquisitions and the study duration.

The 'pre-post' follow-up strategy is one of the simplest and most widely used forms of longitudinal study (Edde et al., 2019, Kolasa et al., 2019, Roy et al., 2021). This study design involves a baseline and a single follow-up MRI acquisition, usually after an intervention (such as treatment or surgery), over different study durations (weeks, months, or years). However, as these studies provide limited estimates of the dynamics of change in MRI parameters, researchers are increasingly adopting 3 timepoints designs: one baseline and two follow-up MRI acquisitions (Bede and Hardiman, 2018, Gregory et al., 2020, Kalra et al., 2020, Tahedl et al., 2021, Van Der Auwera et al., 2021). Some studies include more follow-up acquisitions (>2, Cai et al., 2021, Edde et al., 2023, Koller et al., 2020), but these are still few due to the high costs and subjects’ compliance. Even if multiple study designs are used, the impact of these different follow-up strategies on the results of longitudinal neuroimaging studies remains largely undiscussed. Using a database of multiple MRI acquisitions over time in 36 MS patients (31 relapsing-remitting (RR) MS and 5 secondary progressives (SP) MS), Hu and colleagues showed that lesion evolution indices computed from 4 and 3 MRIs are highly correlated with the lesion evolution index computed from 5 MRIs (Pearson correlation r > 0.9) (Hu et al., 2020). This suggests that a reduced number of MRIs provides a similar lesion evolution index to that obtained with a higher number of MRI acquisitions in MS patients (Hu et al., 2020). In contrast, using between 2 and 6 MRI acquisitions per participant and the FreeSurfer longitudinal pipeline, Beare and colleagues reported an effect of the number of MRIs on the change over time in cortical thickness (Beare et al., 2021). On the other hand, a recent theoretical study showed that the time interval between MRI follow-ups could also have an effect on the results depending on the analytical approach chosen (Müller et al., 2021). Therefore, without providing a clear answer, these studies suggest that the number of acquisitions and the time interval between acquisitions may influence the reported results. However, to our knowledge, no study has evaluated the effect of these factors on quantitative diffusion and myelin-specific MRI data. Yet, measures derived from these two imaging technics are increasingly used in longitudinal studies, especially in MS clinical trials.

Thus, the objective is to evaluate whether and how the number of follow-up examinations, the time interval and the study duration affect the results obtained from quantitative diffusion and myelin-specific MRI data. We used two independent longitudinal MRI datasets, one from healthy participants (n = 20) and the other from patients with relapsing-remitting MS (RR-MS; n = 20), including multi-shell diffusion and inhomogeneous magnetization transfer MRI collected monthly. All subjects were scanned 5 times at four-week intervals (+/-1 week) for a total of 100 MRI examinations per dataset. For each dataset, six different designs were generated. Since longitudinal studies are most often used to predict or assess changes in MRI measures over time and/or to evaluate relationships between MRI parameters and clinical data, the impact of different designs on these two aspects of longitudinal studies were investigated. First, we estimated and compared participant-specific changes in MRI parameters over time between the different designs. In a second, more exploratory analysis using the RR-MS dataset, we explored the potential effect of different designs on the associations between changes in participant-specific MRI measures and changes in clinical data. On the other hand, the longer the study and the more frequent and numerous the MRI acquisitions, the greater the risk that participants will be lost to follow-up. In the case of neurological conditions, possible deterioration of the participant's physical condition during the study, in addition to other socioeconomic factors or risk factors related to the study questions, may contribute to the risk of loss to follow-up. For this reason, we also evaluated the effect of designs on the required sample size.

## 2 Methods

### 2.1 Participants

Longitudinal MRI data from two independent datasets comprising 5 monthly MRI acquisitions are used in this paper. The first dataset was collected from 20 healthy participants and has been previously described in Edde et al, 2023. The second dataset was collected from 20 patients with RR-MS, matched in age and gender with healthy participants, and acquired on the same scanner using a similar protocol to that of the healthy participants. For both datasets, participants were recruited from the CIUSSS de l’Estrie-Centre Hospitalier Universitaire de Sherbrooke (CHUS) and screened for eligibility for MRI, no history of other brain disease or injury, right-handedness, and received financial compensation for their participation. Inclusion and exclusion criteria are detailed in the supplementary data. The study was approved by the Ethics Committee of the CIUSSS de l’Estrie-CHUS in Sherbrooke, Canada, and all participants gave written informed consent prior to enrolment.

No clinical examination was performed in the healthy controls’ dataset, and the characteristics of the participants are shown in Table 1. For the RR-MS dataset, clinical examination was performed by a trained neurology resident (MD) during the day and in a quiet room at baseline (t_0_) and at the last follow-up (t_4_). The clinical assessment included the Expanded Disability Status Scale (EDSS), the sustained attention test using the symbol digit modalities test (SDMT, Benedict et al., 2017), manual dexterity using the Nine-Hole Peg Test (9HPT, Feys et al., 2017) and motor function using the Timed 25-Foot Walk (T25FW, Rudick et al., 1997). Disease duration was estimated from a detailed clinical history (from diagnosis). Changes in the clinical scores were computed for each participant as the difference between the follow-up score and the baseline score (Δscore = score(t_4_) - score(t_0_)). The mean clinical change was computed by averaging the individual changes and is reported in Table 1. Finally, all RR-MS patients were taking medication to treat MS.

**Table 1:** Characteristics of the study participants. Data are presented as mean (standard deviation, SD) or median [range] for continuous variables and count for categorical variables. EDSS: Expanded Disability Status Scale; SDMT: Symbol Digit Modalities Test, 9HPT: Nine Hole Peg Test, T25FW: Timed 25 Foot-Walk; n: number; (s) seconds, (y) years. *Computed as the average of individual changes.

	**Healthy controls’ dataset**	**RR-MS dataset**			***Wilcoxon*** ***p-value***
		Baseline	Follow-up	Change* [range]	
N	20	19	19	19	–
Male/Female (n)	4/16	4/15	–	–	–
Age (y)	36 (4.7)	37.11 (6.8)	–	–	–
Disease duration (y)	–	7 (5.9)	–	–	–
EDSS	–	2 [1 – 6]	2 [1 – 6]	0 [0 – 0.5]	0.4
9HPT (s)	–	23.5 (9.8)	22.7 (6.7)	−0.9 (3.5) [-13.7 – 2.6]	0.28
T25FW (s)	–	6.2 (2.5)	6.1 (2.4)	−0.12 (1.0) [-1.9 – 2.5]	0.6
SDMT (s)	–	57.1 (11.4)	58.4 (9.9)	4.4 (13.8) [-21 – 52]	0.09

### 2.2 MRI data acquisition and processing

For both datasets, whole-brain MRI data were acquired using a clinical 3 T MRI scanner (Ingenia, Philips Healthcare, Best, The Netherlands) with a 32-channel head coil. The MRI datasets consisted of one baseline MRI acquisition (t_0_) and four follow-up MRI acquisitions (t_1→4_) with an interval of 4 weeks (+/- 1 week) between acquisitions over a 6-month period for a total of 100 MRIs per dataset (see supplementary Table 2 for the average number of days). Each MRI acquisition lasted approximately 33 min. For each participant, images were acquired at approximately the same time to avoid potential diurnal effects (i.e., a morning participant had all acquisitions in the morning, with a tolerance of 2 to 3 h). MRI data, including (a) anatomical 3D T1-weighted, (b) multi-shell diffusion-weighted imaging (DWI), (c) inhomogeneous magnetization transfer (ihMT), were acquired according to the same protocol as in Edde et al. 2023. In addition, a fluid-attenuated inversion recovery (FLAIR) image was acquired for each RR-MS patient for lesion segmentation (see supplementary Table 1 for sequencing details and the website for a protocol description, https://high-frequency-mri-database-supplementary.readthedocs.io/en/latest/data/data_description.html#mri-acquisition-parameters).

For the healthy controls’ dataset, processed MRI images from Edde et al., 2023 were used. MRI images from the RR-MS dataset were processed as described by Edde et al., 2023. Briefly, Tractoflow (Theaud et al., 2020, https://github.com/scilus/tractoflow) was used to process each multi-shell diffusion MRI (b at 300, 1000 and 2000 s/mm^2^ for 8, 32 and 60 directions, respectively) and T1w image. This pipeline generates a whole brain tractogram using Ensemble tractography and several diffusion measures, including free water (FW, Daducci et al., 2015, https://github.com/scilus/freewater_flow), DTI, HARDI and NODDI measures (Zhang et al., 2012, https://github.com/scilus/noddi_flow). For the RR-MS dataset, subject-specific lesion masks were added to the TractoFlow input to correct the whole-brain tractograms for lesion burden (see Supplementary data for segmentation procedure). ihMT images were processed using ihMT flow (https://github.com/scilus/ihmt_flow) to generate four myelin-sensitive measures (Varma et al., 2015). For clarity, a subset of MRI measures sensitive to different processes reported in RR-MS was used: extracellular water (free water - FW and isotropic water volume fraction - ISOvf), axonal (total apparent fiber density - AFD total and intracellular volume fraction - ICvf) and myelin content (radial diffusivity - RD and inhomogeneous magnetization transfer (MT) saturation - ihMTdR1sat). Mean Diffusivity (MD) and MT Ratio (MTR) were also included as a reference for clinical trials. The dMRIqc flow (https://github.com/scilus/dmriqc_flow) was used for visual quality assessment of input data and key processing steps. For a complete description of exclusion criteria, see https://high-frequency-mri-database-supplementary.readthedocs.io/en/latest/pipeline/mri_processing.html#quality-control.

### 2.3 White matter virtual dissection

**Fig. 1:**
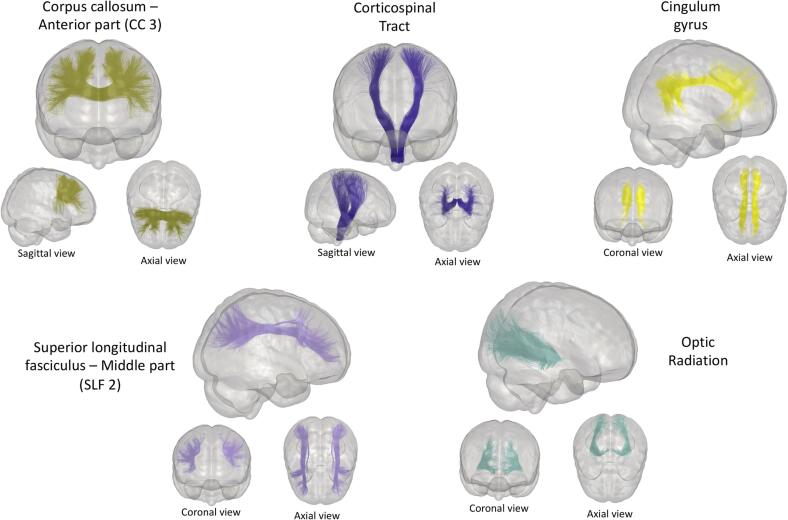
Representation of the selected bundle models used by RecobundlesX as shape priors to extract the bundles from the whole tractogram. Bundles from both hemispheres are shown and displayed on the glass brain.

Major white matter fascicles were automatically extracted using RecoBundlesX (Rheault et al., 2019, https://zenodo.org/record/4104300#.YNoP1XVKiiM), a multi-atlas/multi-parameter version of RecoBundles (Garyfallidis et al., 2018). Bundles were corrected for white matter lesions (i.e., streamlines crossing white matter lesions are excluded). Thus, data analyses were performed only on normal-appearing white matter (NAWM). For clarity, results are reported for a subset of bundles known to be affected in RR-MS patients (Fig. 1). The anterior part of the corpus callosum (CC3), corticospinal tract (CST), cingulate gyrus (CG), middle superior longitudinal fasciculus (SLF2) and optic radiation (OR) were selected as bundles of interest (Catani and de Schotten, 2012). Finally, average diffusion and myelin measurements were extracted for each bundle. Bundle colors were matched throughout the results. Visual quality was assessed using dMRIqc flow (https://github.com/scilus/dmriqc_flow).

## 3 Experimental designs

**Fig. 2:**
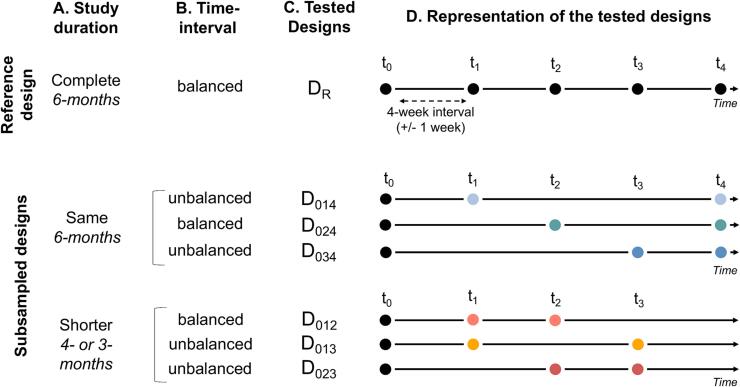
Representation of the reference design and tested designs generated. The reference design (D_R_) refers to 5 MRI acquisitions. The tested models refer to the different organizations of the two follow-up MRI acquisitions over time (subsampled designs). A) the two study duration options, B) the two time-interval options, C) the nomenclature of the designs, and D) the visual representation of the follow-ups. Each dot represents one MRI acquisition at one study time. Design colors are matched throughout the results.

Based on the common case of one baseline and two follow-up acquisitions (3 MRI acquisitions), 6 different designs were generated by varying the number of follow-up acquisitions, the time interval between them, and the study duration (Fig. 2, see also supplementary Table 2). The following nomenclature will be matched throughout the paper: D for Design, the indices 0,1,2,3,4 associated with D correspond to the MRI acquisition number and the index R corresponds to the Reference Design (D_R_, see Fig. 2C). For both datasets, the 5 MRI acquisitions were used as the reference design (D_R_). For all tested designs, the baseline MRI (t_0_) is always identical, but the MRI follow-up acquisition (t_1→4_) varies over two study durations (Fig. 2A):

- over the same study duration as the reference design (i.e., 6 months: D_014_, D_024_, D_034_, blue spectral colors).
- over a shorter study duration (i.e., one 3-month design: D_012_ and two 4-month designs: D_013_ and D_023_, red spectral colors).

For each study duration, two time interval strategies are tested (Fig. 2B):

- balanced time intervals (i.e., equal time intervals between MRI acquisitions: D_024_ and D_012_)
- unbalanced time intervals (i.e., different time intervals between the baseline and the first follow-up MRI can be short: D_014_ and D_013_, or long: D_034_ and D_023_).

For each tested design, Fig. 2C provides the corresponding nomenclature and Fig. 2D provides a visual representation of the follow-up.

### 3.1 Sample sizes estimation

For both datasets, the sample size for the reference design and each tested design was estimated using G*Power 3.1 (Faul et al., 2009). We considered the minimum number of subjects required to achieve a statistical power of 0.8 and significance of alpha = 0.05 for Group (1) × Time (5) within groups ANOVA for the reference design (D_R_, i.e., to detect a difference in the longitudinal course of a measure) and Group (1) × Time (3) within groups ANOVA for the 6 tested designs. A small effect size (f = 0.2) was used for all measures and bundles (De Santis et al., 2019, Feinstein et al., 2020, Weber et al., 2022). Pearson correlation coefficients were used to account for the correlation between repeated MRI measures (across subjects and acquisitions) using the bundle averages of diffusion and myelin measures corresponding to MRI acquisitions of different designs.

### 3.2 Data analysis

We assessed changes over time in the clinical data of RR-MS patients using the Wilcoxon paired test with a significance level of p < 0.05. Next, the impact of the tested designs was evaluated on 1) the changes in MRI parameters over time for each individual (defined as ‘change over time’) and 2) the associations with changes in clinical outcomes. The same methodology was applied to the reference design and the 6 tested designs for both analyses. Statistical analyses were performed using R v.4.2 (Foundation for statistical computing, Vienna; packages lmer, rstatix and boot).

First, the effect of designs on the changes in MRI parameters over time was done on the healthy controls’ and RR-MS datasets, independently. The analysis of the healthy dataset will serve as a reference for the pathological population to ensure that tested design differences are not associated with variability due to repeated measurements and image processing. To assess changes in MRI parameters over time across bundles, we used a linear mixed model, which is widely used when data are not independent and can also account for the influence of different sources of variability. The model considered time as a fixed effect and participant as a random effect, so that each participant's data was fitted with a unique intercept and slope. Age, sex, and disease duration for the RR-MS dataset at baseline were included as fixed effects in the model to adjust for potential confounding effects. The resulting slope for each participant corresponds to the change in each MRI parameter over time. Participant-specific changes from each design (reference and tested) were extracted and compared with repeated measures analysis of variance (ANOVA). Data were tested for sphericity using Mauchly's test. In cases where the sphericity assumption was not met, a Greenhouse-Geisser correction for degrees of freedom was applied. The post-hoc Tukey test was used to assess specific differences between designs. The significance level was set at p < 0.05. Benjamini-Hochberg false discovery rate (FDR, Benjamini and Hochberg, 1995) correction was used to adjust the significance for multiple comparisons (p < 0.05). In addition, to assess the similarity of the changes over time resulting from each design, the Pearson correlation between each possible pair of designs was used for each MRI measurement.

Second, a more exploratory approach was used to assess the effect of designs on the associations between changes in MRI parameters and changes in clinical outcomes. Since no clinical examination was performed in the healthy controls’ dataset, only the RR-MS dataset was used for the correlation part. To account for the non-Gaussian distribution of the clinical outcomes, Spearman correlation coefficients were used to estimate the associations between changes in MRI parameters obtained from the tested designs and changes in clinical outcomes (Δscore = score (t_4_) - score (t_0_)). Four scores widely used in MS were selected: EDSS, SDMT, 9HPT and T25FW. Associations with the 6 tested designs are shown only if an association was already present with the reference design (defined as ‘preserved associations’). Significant associations with the 6 tested designs that were not present with the reference design are reported in the supplementary data (defined as ‘new associations’). Because the objective is to determine the ability of the tested designs to preserve an existing association with the reference design in the same population, no clinical interpretation of the associations was made and no correction for multiple comparisons was applied. To compensate, a p < 0.01 was considered to indicate a significant association.

## 4 Results

### 4.1 Demographic and clinical characteristics at baseline and follow-up

Table 1 shows baseline, follow-up and change in clinical data for the datasets. In the RR-MS dataset, the median EDSS at baseline was 2.0 and the median disease duration was 7 years. One subject who relapsed during the study period was excluded. There were no significant changes in clinical scores during follow-up.

### 4.2 Impact of designs on the healthy controls’ dataset

#### 4.2.1 Impact on change in time for the healthy controls’ dataset

Changes in MRI parameters over time between the tested designs and the reference design were similar for the healthy controls’ dataset. A good (D012 [r = 0.68]) to very good (D034 [r = 0.89]) similarity is found for changes in MRI parameters over time with the tested designs compared to the reference design (DR), regardless of the bundles and MRI measures (supplementary Table 4). An equivalent similarity is found across bundles (Pearson coefficient ranging from 0.76 to 0.84, supplementary Table 4). However, the level of similarity is more variable for the MRI measures. ISOvf shows the lowest level of similarity (r = 0.68), while AFD total shows the highest level on average (r = 0.93) (supplementary Table 4).

#### 4.2.2 Sample size estimation in the healthy controls’ dataset

**Fig. 3:**
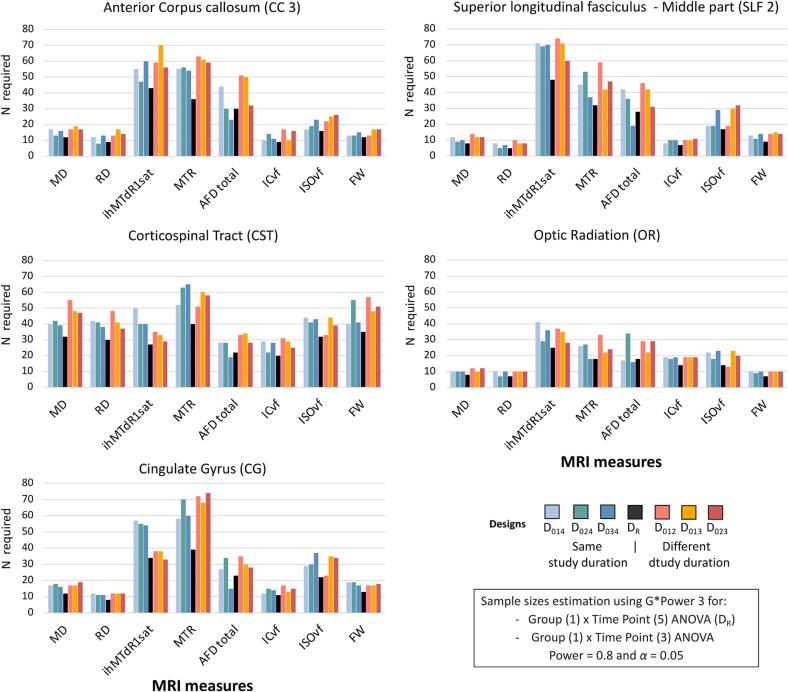
Estimated sample sizes in the healthy controls’ dataset for the reference design and each tested design to achieve 0.8 power and α = 0.05 in bundles for a Group (1) × Time (3 or 5) ANOVA. Sample size requirements for each bundle and design were estimated using the Pearson correlation coefficients between repeated MRI measures (across subjects and (3 or 5) acquisitions) using the bundle averages of MRI measures corresponding to MRI acquisitions of different designs.

The number of participants required for the tested designs (n = 27 to 29 on average) is higher than for the reference design (D_R_, n = 21 on average, Fig. 3, supplementary Table 5). In contrast, the number of participants required across the tested designs is relatively similar regardless of study duration and time interval, although there is some variability. Measures derived from DTI require the fewest participants in most bundles: MD (n ∼ 20), RD (n ∼ 16), and FW (n ∼ 21). However, the measures derived from advanced diffusion and magnetization transfer images (MTI) require more participants (e.g., ihMTdR1sat [n ∼ 47], MTR [n ∼ 49] and AFD total [n ∼ 30]; see supplementary Table 5). This heterogeneity in sample size estimation is found for all bundles except CST. The number of subjects required for the CST-derived measures is higher for all MRI measures, ranging from 20 (AFD total) to 65 (MTR; n = 39 on average), and even for the DTI-derived measures (MD [n ∼ 43] and RD [n ∼ 40]). The other bundles had a similar average number of subjects required (CC3 and CG [n ∼ 28], OR [n ∼ 18], SLF2 [n ∼ 26], supplementary Table 5).

### 4.3 Impact of designs on RR-MS dataset

#### 4.3.1 Impact on changes in time of MRI parameters in RR-MS dataset

**Fig. 4:**
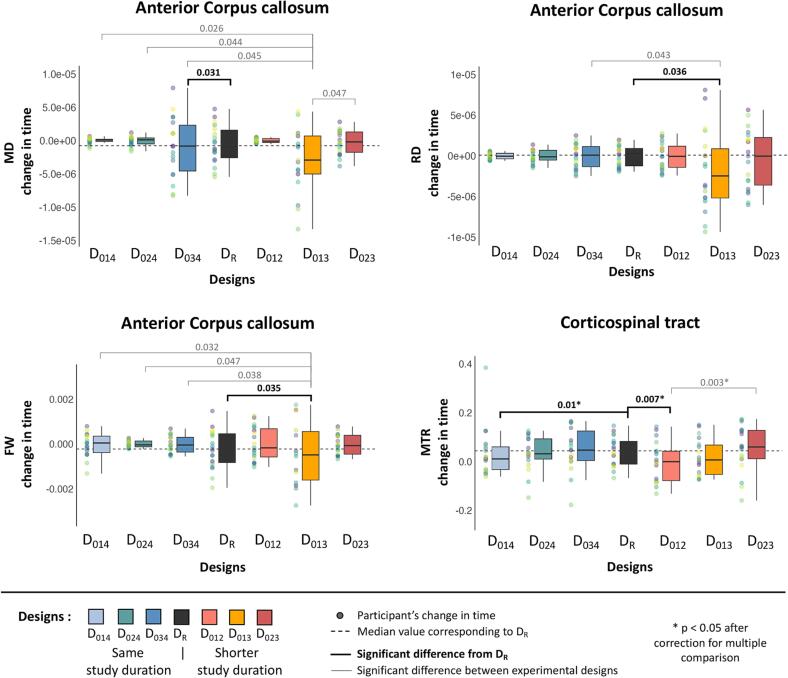
Boxplot of the changes for the reference design and tested designs for the RR-MS dataset. Each point on the left of the boxplots represents a participant's change with the color corresponding to each participant. This shows the impacts of tested designs on the participants' change measures. Displayed are the respective changes in MRI parameters over time for the reference design (black boxplot, D_R_) and design over the same study duration on the left (blue color spectrum, D_014_, D_024_, D_034_) and over shorter study durations on the right (red color spectrum, D_012_, D_013_, D_023_). The black dotted horizontal line corresponds to the median of the D_R_, the box represents the upper and lower quartiles, and the whiskers represent the 1.5-fold IQR. Bold lines correspond to significant post-hoc differences from the D_R_. Grey lines correspond to significant post-hoc differences between the tested designs. * p < 0.05 after correction for multiple comparisons. (For interpretation of the references to color in this figure legend, the reader is referred to the web version of this article.)

Differences in changes over time between the tested designs and the reference design were found for CC3-RD, CC3-MD, CC3-FW and CST-MTR (i.e., 4 of the 40 ANOVA comparisons). Changes over time in CC3-MD and CST-MTR were significantly different from the reference design (D_R_) for the designs over the same study duration (6 months) and an unbalanced time interval D_034_ and D_014_, respectively (Fig. 4, bold black line). Changes over time in CC3-RD and CC3-FW, and CST-MTR differed for shorter study duration designs D_013_ and D_012_, respectively (Fig. 4, bold black line). The post-hoc analyses also showed more frequent differences between the tested designs (Fig. 4, grey line). Changes over time in CC3-MD, -RD and -FW differed significantly from the shorter study duration design D_013_ for most of the other tested designs; similarly, change over time in CST-MTR differed significantly between two shorter study duration designs D_012_ and D_023_ (Fig. 4, grey line). However, only differences for changes in CST-MTR remain after correction for multiple comparisons. No difference is observed for the other bundles.

Note that the distribution of changes over time of the tested designs is more or less variable compared to the distribution observed with the reference design (D_R_). Compared to the reference design (D_R_, range MD = 0.20, RD = 0.19 expressed as x10^-4^ and FW = 0.006), the design with the shorter study duration D_013_ has a wider distribution for CC 3 MRI measurements (range MD = 0.49, RD = 0.43 expressed as x10^-4^ and FW = 0.013), whereas the designs with the same study duration D_014_ or D_024_ have a narrower distribution (range MD = 0.018, RD = 0.015 expressed as x10^-4^ and FW = 0.003; range MD = 0.028, RD = 0.035 expressed as x10^-4^ and FW = 0.0005, respectively, Fig. 4).

Despite these detectable differences, Pearson correlations between reference design and tested designs show good (D012 [r = 0.62]) to very good (D034 [r = 0.87]) agreement between changes over time, regardless of bundle and MRI measures (supplementary Table 6). An equivalent similarity is found across bundles (Pearson coefficient ranging from 0.72 to 0.85, supplementary Table 6). However, the level of similarity is more variable for the MRI measures, with RD showing the lowest level of similarity (r = 0.5), while ICvf shows the highest level (r = 0.89) on average (supplementary Table 6).

#### 4.3.2 Sample sizes estimation in RR-MS dataset

**Fig. 5:**
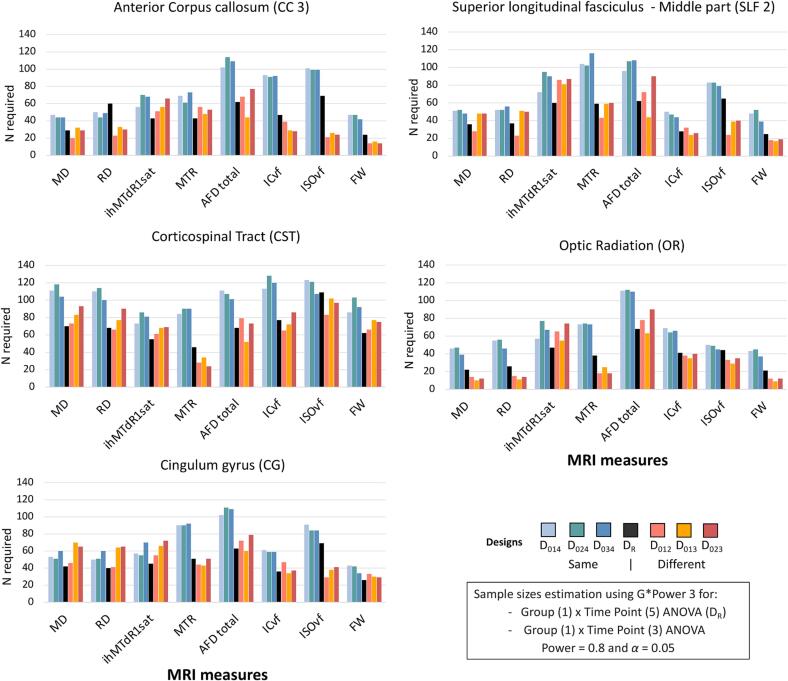
Estimated sample sizes in RR-MS dataset for the reference design and tested design to achieve 0.8 power and α = 0.05 in bundles for a Group (1) × Time (3 or 5) ANOVA. Sample size requirements for each bundle and design were estimated using the Pearson correlation coefficients between repeated MRI measures (across subjects and (3 or 5) acquisitions) using the bundle averages of MRI measures corresponding to MRI acquisitions of different designs.

Regarding sample size estimation, the number of participants required for the tested designs (n = 45–77 on average) is generally higher than for the reference design (D_R_, n = 50 on average, Fig. 5, supplementary Table 7). The number of subjects required varies by tested design, the same study duration designs require more subjects (n ∼ 76) than those on shorter study durations (n ∼ 48, Fig. 5). FW is the MRI measure requiring the fewest subjects (n ∼ 40), followed by DTI-derived measurements in most bundles with MD (n ∼ 51) and RD (n ∼ 52). In contrast, the advanced diffusion and MTI-derived measures require more participants (n > 55), including AFD total (n ∼ 85), ihMTdR1sat (n ∼ 67) and ISOvf (n ∼ 66; see supplementary Table 7). This heterogeneity in sample size estimation is found for all the bundles except CST (Fig. 5). The number of subjects required for CST-derived measures is higher, ranging from 56 (MTR) to 106 (ISOvf; n = 84 on average), including DTI-derived measures (MD [n = 93] and RD [n = 90]). The other bundles had a similar average number of subjects required (CC3 [n ∼ 53], CG [n ∼ 58], OR [n ∼ 47] and SLF2 [n ∼ 57], supplementary Table 7).

#### 4.3.3 Impact on associations with changes in clinical outcomes

**Fig. 6:**
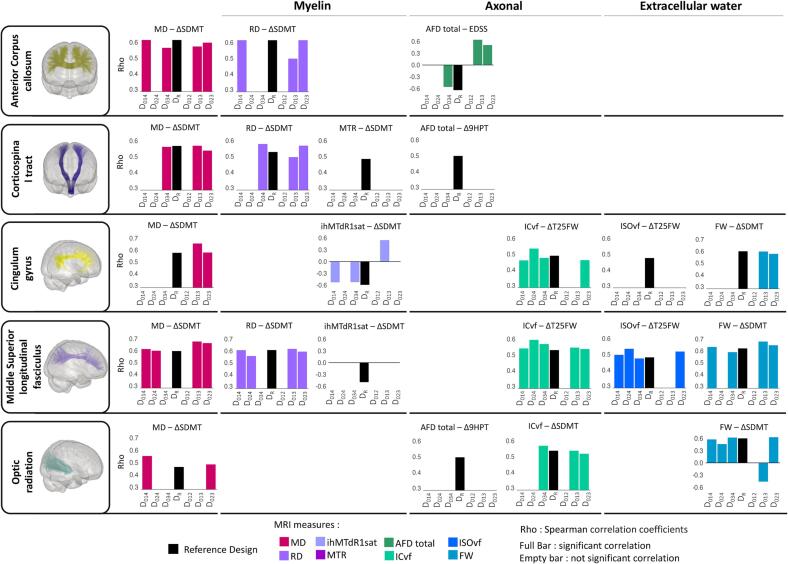
Spearman's correlation coefficient between changes in clinical outcomes and MRI measures in RR-MS dataset. Each bar shows Spearman's correlation coefficient (Rho) for each design and bundle. The black bars correspond to the reference design (D_R_). The other bars correspond to the tested designs and the colors correspond to the MRI parameters. Presence of a bar: correlation is present and significant (p < 0.01). Absence of bar: correlation is present but is not significant; for better visualization, the correlation value was set to 0.

Changes in MRI parameters from the reference design (D_R_) were associated with at least one of the changes in clinical scores for most bundles (black bar, Fig. 6). Compared to the reference design (D_R_), associations with changes in clinical outcomes are preserved for most of the tested designs. However, for some bundles and MRI measures, no associations are preserved regardless of the designs tested (CST MTR, AFD total; CG ISOvf; middle SLF ihMTdR1sat; OR AFD total, Fig. 6). On the other hand, some associations with changes over time of designs with shorter study duration (D_013_, D_023_) exhibit an inverse correlation compared to the reference design (CC3 AFD total, D_013_, D_023_; CG ihMTdR1sat, D_013_; OR FW, D_013_, Fig. 6). No significant correlations emerged between changes in clinical scores and the shortest study duration design (D_012_, Fig. 6).

**Fig. 7:**
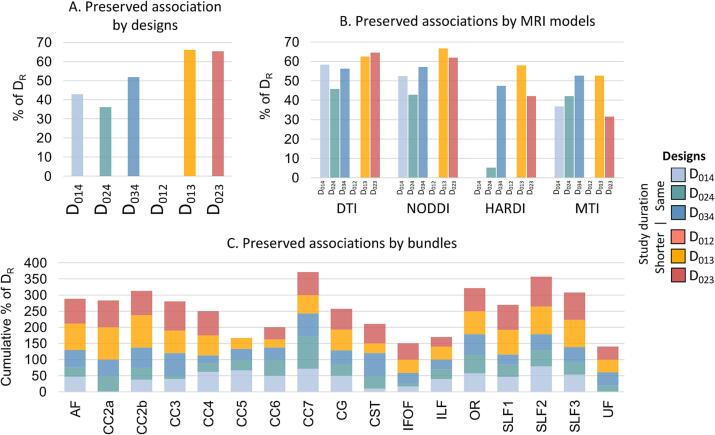
Frequency of preserved association in RR-MS dataset. The bars are expressed as a percentage of the number of associations in the reference design (D_R_). A) Bars represent the frequency of preserved associations for each design and B) for each MRI model. C) Stacked bars represent the cumulative frequency of preserved associations for each bundle and each design. The color represents the design.

The frequency of preserved associations per bundle and MRI measure was computed over all available bundles and MRI measures to avoid the bias associated with our selection and is expressed as a percentage of the total association of the reference design (D_R_) total association (total number of associations = 133). The frequency of preserved associations is high for tested designs with an unbalanced time interval (>50 % of D_R_ association), especially those with shorter study duration (D_023_: 64.6 % and D_013_: 62.5 %), followed by those with the same study duration (D_014_: 58.3 % and D_034_: 56.3 %). Tested designs with a balanced time interval are the least frequent with a preserved association frequency of 45.9 % for D_024_ while D_012_ is never represented Fig. 7A. The DTI and NODDI-derived measures have a similar frequency of preserved associations with a percentage consistently above 40 % regardless of the tested designs and up to 60 % for tested designs over shorter study duration (D_013_ and D_023_, Fig. 7B). MTI-derived measures are more variable with a frequency of > 30 % for all tested designs and up to > 50 % for the unbalanced designs with shorter (D_013_) and the same study duration (D_034_). Finally, HARDI-derived measures are the least consistent, with the same study duration designs D_014_ and D_024_ that are almost absent (<5%). The frequency of preserved associations is also heterogeneous across bundles. The CC7, the middle and lower parts of SLF (SLF2 and SLF3), OR and CC 2a bundles show the highest frequencies of preserved associations (>50 % on average) and conversely for CC 5, IFOF, ILF or UF (<30 % on average, Fig. 7C).

Note that new associations (i.e., not found for the reference design) are found for all tested designs except for the shortest tested design D_012_. The shorter study duration designs D_013_ and D_023_ have the highest frequency of new associations (number of new associations ≥ 60) compared to the others (n < 50). The CC 5, CST, ILF and UF bundles (n > 4 on average), and the MTI-derived measures (n ∼ 12 on average) also have higher frequencies compared to other bundles (n ≤ 3 on average) and MRI designs (n ≤ 8 on average; Supplementary Fig. 1, and supplementary Table 8).

For a summary of the main observations, see Table 2.

**Table 2:** Summary of the results corresponding to the evaluated analyses and designs.

**Parameters**	**Number of follow-ups**	**Time-interval**	**Study duration**
Changes over time	3 MRI acquisitions similar to 5 MRI acquisitions	Unbalanced design is more consistent	Shorter study duration similar to longer study duration
Sample size	Higher with 3 MRI acquisitions	Low impact	Higher N for a longer study duration
Associations	Similar to 5 MRI acquisitions May lead to a lack of association	Similar to 5 MRI acquisitions Unbalanced design: time intervals depend on the dynamics of change in the target population	Similar to 5 MRI acquisitions A shorter study duration could be misleading

## 5 Discussion

This study used two independent datasets, acquired using a similar protocol and comprising 5 monthly MRI acquisitions from 20 healthy volunteers and 20 patients with RR-MS. The aim was to assess whether and how the number of follow-up acquisitions, the time interval and the study duration affected the quantitative diffusion and myelin-specific MRI data results. Six designs were generated from the 5 MRI acquisitions by varying the number of follow-ups, the time interval between them, and the study duration. Tested designs were compared for 1) changes in MRI measures over time, 2) sample size estimates required for a group (1) × time (3) or (5) ANOVA to achieve statistical power of 0.8 and significance of α = 0.05 for a small effect size, and 3) associations between changes in MRI measures and change in clinical data of RR-MS dataset.

Changes over time obtained from 3 MRI acquisitions are similar to those obtained from 5 MRI acquisitions, suggesting that a reduced number of MRI acquisitions provides a similar estimate of changes over time. In addition, good to very good similarity was also found for the changes over time obtained from the reference design and the tested designs (Pearson coefficient r between 0.6 and 0.8 on average). This is consistent with recent studies showing that fewer MRI acquisitions yield results consistent with those obtained with a higher number of MRI acquisitions (Beare et al., 2021, Hu et al., 2020). For the RR-MS dataset, some differences are found when comparing changes over time in the anterior corpus callosum (CC3) and corticospinal tract (CST) bundles for some MRI measures over time. This bundle- and measure-dependent effect is not surprising given the bundle- and measure-specific variability of MRI measures described previously (Edde et al., 2023). In contrast, in the healthy controls’ dataset, no significant differences are found, and a very high similarity (r = 0.8 on average) is observed between the changes obtained with the reference design and those obtained with the tested designs over time. This suggests that the differences observed in the RR-MS dataset are population-specific and not the result of variability related to the repetition of measurements over time or random effects of measurement errors such as image noise or MRI signal variation (Wang et al., 2012). On the other hand, it should be noted that the lesion intensity profiles of Hu and colleagues illustrate an important aspect of the reduced number of MRI acquisitions (Hu et al., 2020). The authors show that a high number of MRI acquisitions produces accurate lesion intensity profiles characterized by a progressive decrease in MRI parameters over time, followed by a plateau (Hu et al., 2020). With 3 MRI acquisitions, the profiles are less accurate but capture the initial decrease and plateau, whereas, with 2 MRI acquisitions, the lesion intensity profiles show only a linear decrease over time, masking the initial decrease (Hu et al., 2020). Therefore, although the conclusion is similar, a smaller number of MRI acquisitions may result in ignoring some of the dynamics of changes over time in the parameters being studied. Taken together, this suggests that the need for frequent measurements depends on the purpose of the study and the accuracy of the dynamics required by the condition being studied.

Designs with an unbalanced time interval showed the highest similarity (r > 0.8), regardless of study duration. In contrast, a balanced time interval gives less consistent results (D_024_, D_012_), especially for a very short study duration (D_012_, r < 0.7). This effect of the time interval is consistent with the theoretical study by Müller et al., 2021. Using different analytical approaches to estimate linear changes in simulated striatal atrophy over time, Müller and colleagues show that for 3 MRI acquisitions over time, an identical time interval between the three measurements would render the intermediate measurement useless, while an unbalanced time interval is more relevant, particularly in the case of a short interval between two MRI acquisitions (Müller et al., 2021). Thus, the choice of the time interval could be based on the assumption of the dynamics of change over time. A baseline measurement with a short time interval for the first follow-up and a second follow-up with a longer time interval (i.e., D_014_, D_013_) is an option if the aim is to assess a more or less rapid effect on brain structure, e.g., the effect of a treatment. A baseline measurement with a long time interval for the first follow-up and a second follow-up with shorter time interval that could be used to confirm the observations (i.e., D_034_, D_023_) is an option if the aim is to evaluate an effect that will take longer to affect brain structure, e.g., the effects of therapeutic intervention or long-term treatment. Therefore, similar to the number of MRI acquisitions, the optimal time interval will depend on the assumption of the dynamics of change in the target population (either due to the natural course or due to an intervention).

On the other hand, the analyses also show that variation in a time interval and study duration leads to a more variable distribution of changes at the individual level. These differences in distribution are reflected in the ranges of measurements, suggesting a different variability of measurements for a reduced number of MRI acquisitions. This also affects the correlation between MRI measures over time and thereby the sample sizes required. In RR-MS patients, the sample sizes required for the same study duration are larger (n ∼ 76) than those required for a shorter study duration (n ∼ 48), without being affected by the different time intervals. This suggests that study duration has a greater impact on the required sample size, as a longer study a priori requires more subjects than a shorter study. In addition, the number of subjects also varies according to the MRI measures, with DTI and FW-derived measures requiring fewer subjects (n ∼ 50) than measures derived from advanced diffusion or myelin designs (n > 50). Most bundles showed a common pattern. In contrast, the CST bundle had a pattern in which the diffusion measures required larger sample sizes (n ∼ 84) than the other measures, which required the same or smaller sample sizes (n ∼ 54). To date, we have not found a valid explanation for this observation. All we can say is that it doesn't seem to be specific to the RR-MS population, since the healthy subjects show a similar pattern. This variability in the number of subjects required by bundles and MRI measures was also found in the healthy controls' dataset, although the overall number of subjects was lower (n < 30). However, the number of subjects required in the healthy dataset is little affected by study duration or time interval, suggesting some independence from these two factors. Again, this is not surprising given the bundle- and measure-specific variability of MRI measurements described previously (Edde et al., 2023) and is consistent with a recent study also showing heterogeneity between measurements and MRI bundles considered (Koller et al., 2020).

In the RR-MS dataset, the associations with clinical data show that most of the designs tested provide consistent results with the reference designs, supporting the previous observations. The variability across bundles and MRI measures described above is also found for associations with clinical data, with MTI and HARDI-derived measures appearing to be more sensitive to design than DTI and NODDI, and CC 5, IFOF, ILF or UF bundles being more sensitive than CC7, SLF, OR and CC 2a bundles. Again, the strongest consistency is observed when unbalanced designs are used, as opposed to balanced designs, regardless of study duration. Hu and colleagues, who also examine the relationship between the lesion evolution index and indices of therapeutic intervention, show that the relationships are in the same direction with fewer MRI acquisitions than with all MRI acquisitions, although the effect sizes are smaller (Hu et al., 2020). However, two types of inconsistency are observed: finding an association in the opposite direction to that found with the 5 MRI acquisitions, and not finding the association observed with 5 MRI acquisitions. The case of an association in the opposite direction is found only for two shorter study duration designs, D_013_ and D_023,_ and represents only 4.1 % of the total associations. Furthermore, correction for multiple comparisons would probably minimize this inconsistency, which is not the case here. Note that the follow-up of the clinical outcome corresponds to the last follow-up of the reference data, i.e., time point number 4, so we cannot ignore that an evaluation of the clinical outcome corresponding to the last point of the designs over a shorter study duration, i.e., time point number 3, would not give rise to such inconsistencies. On the other hand, the failure to find an association between changes in MRI parameters and changes in clinical outcomes in some of the tested designs suggests that many MRI acquisitions are more sensitive than a limited number of MRI acquisitions (regardless of study duration). However, given the cost and increased burden on study participants of collecting large numbers of data, it is important to consider that improved sensitivity could be achieved with more participants. These results show that differences in individual changes in MRI parameters over time, depending on the follow-up strategy, could affect the associations with clinical outcomes and could even be misleading. Together, this suggests that design strategies may influence the sensitivity and specificity of associations with clinical outcomes. Aware of the limitations of clinical assessment associated with the short follow-up period, the impact on MRI-clinical associations remained deliberately exploratory. That's why it's important to keep in mind that associations with clinical changes may not be fully meaningful and should be used with caution.

It is also important to consider these results regarding the statistical approach used. Indeed, the linear mixed model approach is used because of its applicability (Bernal-Rusiel et al., 2013, Rencher and Schaalje, 2008) and its use in several longitudinal studies (De Meo et al., 2021, Dennis et al., 2019, Greeke et al., 2017, He et al., 2020). Other fitting models are available (Herle et al., 2020), such as the least shrinkage and absolute selection operator (Lasso, Bose et al., 2022, Tibshirani, 1996), growth mixing models (Kwon et al., 2021, McArdle and Epstein, 1987) or the generalized equation estimation (Liang and Zeger, 1986, Shahrbanian et al., 2019). However, these models are generally oversized to solve for linear modelling of three data points. Furthermore, although the choice of statistical model and the way in which different variables are included can influence changes over time (Kwon et al., 2021), there is currently no consensus in the research community on standard models for specific questions. Finally, as we already mentioned, we have examined the influence of acquisition interval and study duration using data from an existing study with a short study duration (6 months) and a very selective sample. This is a relatively short period for monitoring the evolution of neurological conditions and the clinical course of patients, with most clinical trials and longitudinal studies having a duration of 1–2 years and more. To overcome this limitation and go further, patients will be followed up two years after the start of the study to assess the longer-term evolution of the different clinical and MRI measures used. Although this may not be fully generalizable and not resolve the general issue of determining the appropriate number of longitudinal measurements needed to assess the evolution of MRI parameters and the relationships with the evolution of pathology at the clinical level, we provide insight into the influence of design in a pathological population. Indeed, it is important to consider these results in the context of the population and the MRI and clinical parameters studied. The evolution of the RR-MS subjects over time influences how the time interval and study duration can account for brain changes over time. In our case, the subjects are stable (no change in EDSS), under treatments and show little variability over the study duration. However, participants in clinical trials involving an intervention (treatment or other) may exhibit different dynamics. Along the same line, studies of other MRI variables, whose behavior is more stationary or time-varying, may lead to different considerations.

Based on our results, 3 MRI acquisitions with an unbalanced time interval might be sufficient to estimate changes over time in the pathological populations. The study duration and the organization of the unbalanced time intervals depend on the hypothesis of the dynamics of change in the target population. The sample size should consider the study duration and be sufficient to have the necessary sensitivity and specificity for associations with clinical parameters. Finally, all of these will depend on the white matter bundle and MRI measures considered.

## 6 Conclusion

We showed that the number of MRI acquisitions, the time interval between acquisitions, and the study duration affect the results obtained from quantitative diffusion and myelin-specific MRI data. A reduction from 5 to 3 MRI acquisitions with unbalanced time intervals may be sufficient to assess changes over time in healthy controls and RR-MS datasets. The improvement provided by more acquisitions may be negligible if the objective is to estimate changes in brain structure over time. However, the assumption of the dynamics of change in the target population and the accuracy required to capture these dynamics are critical to the choice of number and time interval of MRI acquisitions. In addition, a more moderate consistency for associations shows that the sensitivity and specificity of associations with clinical outcomes are affected by study design. A larger sample size would improve sensitivity, considering that the number of participants is affected by the study duration in the RR-MS dataset; a longer study requires more subjects than a shorter study. Note that all these factors vary depending on the bundles examined and the MRI measures considered. Because the design does not affect the healthy control dataset, the differences reported in RR-MS are population-specific and not due to other sources of variation. Thus, this study provides insight into the effect of follow-up strategy in a longitudinal study of a healthy and pathologic population and suggests strategies that could be applied in clinical trials.

**Code availability**

All Nextflow and code used to process the T1 and DWI images, ihMT images, RecoBundlesX and Tractometry are available at https://github.com/scilus.

## Funding

Part of this research was supported by the NSERC Discovery grant (www.nserc-crsng.gc.ca), the Université de Sherbrooke Institutional Chair in Neuroinformatics from Pr Descoteaux (www.usherbrooke.ca) and Mitacs Accelerate program (www.mitacs.ca). The funders had no role in study design, data collection and analysis, the decision to publish, or the preparation of the manuscript.

## CRediT authorship contribution statement

**Manon Edde:** Conceptualization, Data curation, Formal analysis, Methodology, Visualization, Project administration, Writing – original draft, Writing – review & editing. **Francis Houde:** Conceptualization, Visualization, Formal analysis, Writing – review & editing. **Guillaume Theaud:** Resources, Data curation, Writing – review & editing. **Matthieu Dumont:** Resources, Data curation, Writing – review & editing. **Guillaume Gilbert:** Methodology, Resources, Writing – review & editing. **Jean-Christophe Houde:** Resources, Data curation, Writing – review & editing. **Loïka Maltais:** Resources, Data curation, Writing – review & editing. **Antoine Théberge:** Conceptualization, Data curation, Project administration, Writing – review & editing. **Moussa Doumbia:** Data curation, Validation, Writing – review & editing. **Ann-Marie Beaudoin:** Conceptualization, Writing – review & editing. **Emmanuelle Lapointe:** Project administration, Validation, Writing – review & editing. **Muhamed Barakovic:** Writing – review & editing. **Stefano Magon:** Writing – review & editing, Conceptualization, Supervision. **Maxime Descoteaux:** Conceptualization, Supervision, Funding acquisition, Methodology, Writing – review & editing.

## Declaration of Competing Interest

The authors declare that they have no known competing financial interests or personal relationships that could have appeared to influence the work reported in this paper.

## Data Availability

Data will be made available on request.
