# Supplementary material for: Impact of follow ups, time interval and study duration in diffusion & myelin MRI clinical study in MS
Source: Neuroimage Clin. 2023 Oct 12;40:103529. doi: 10.1016/j.nicl.2023.103529 (PMC10591008; doi:10.1016/j.nicl.2023.103529)
Supplement: Supplementary data 1 [file mmc1.docx]

**Impact of follow ups, time interval and study duration in diffusion & myelin MRI clinical study in RR-MS**

Manon Edde^1,2^, Francis Houde^1^, Guillaume Theaud^1^, Matthieu Dumont^1^, Guillaume Gilbert^3^, Jean-Christophe Houde^1^, Loïka Maltais^1^, Moussa Doumbia^4^, Ann-Marie Beaudoin^2,4^, Emmanuelle Lapointe^4^, Muhamed Barakovic^5^, Stefano Magon^5*^, and Maxime Descoteaux^1,2*^

^1^ Imeka Solutions, Inc., Sherbrooke, QC, Canada.

^2^ Université de Sherbrooke, Sherbrooke, QC, Canada.

^3^ MR Clinical Science, Philips Healthcare Canada, Mississauga, Ontario, Canada,

^4^ Université de Sherbrooke, CIUSSS de l’Estrie-CHUS Fleurimont, Sherbrooke, QC, Canada.

^5^ Roche Pharma Research and Early Development, Neuroscience and Rare Diseases, Roche Innovation Center Basel Switzerland, F. Hoffmann-La Roche Ltd., Basel, Switzerland

* Co-senior authors

**Corresponding author:**

Manon Edde, Université de Sherbrooke, 2500, boul. de l'Université, Sherbrooke (Québec) J1K 2R1, Canada, eddemanon@gmail.com

**Method supplementary**

1. **Study enrolment criteria**

**Inclusion Criteria**

- Ability to provide informed consent and fully understand instructions;
- Age between 18 and 55 years;
- Diagnosis of RRMS confirmed by a neurologist less than 15 years ago;
- Expanded Disability Status Scale (EDSS) score between 0 and 6;
- Stable immunomodulatory therapy for MS for 3 months prior to study enrollment.

**Exclusion criteria**

- Progressive form of MS (primary or secondary);
- Suspected clinical progression in the year prior to study entry;
- Current pregnancy or expected pregnancy within the next year;
- Treatment with intravenous corticosteroids (Solumedrol) in the 30 days prior to study entry;
- Known history of CNS pathology other than MS;
- Claustrophobia or other reason that would prevent the subject from tolerating the imaging study;
- Presence of a metal object, medical or otherwise, in the patient's body.

1. **Clinic assessment**

The Expanded Disability Status Scale (EDSS), which rates neurological impairment, was scored as per individual study protocol. The T25FW has established reliability (Rosti-Otajärvi et al., 2008) and validity (Kieseier and Pozzilli, 2012) and is commonly used in MS clinical trials (Polman and Rudick, 2010). Participants were instructed to walk at their quickest, safe speed along a flat 25-foot walkway (Rudick et al., 1997). The average time of two trials was used as the final score.

1. **MRI data acquisition parameters**

All MRI acquisition parameters are detailed in Table 1.

(a) 3D T1-weighted MPRAGE image was acquired axially at 1.0 mm isotropic resolution, repetition time (TR)/echo time (TE)= 7.9/3.5ms, field-of-view (FOV) = 224x224 mm^2^ yielding 150 slices, flip angle = 8° for an acquisition time of 4 min 20 s.

(b) Multi-shell DWI images were acquired with a single-shot EPI spin-echo sequence at 2.0 mm isotropic resolution, TR/ TE= 4800/92 ms, SENSE factor = 1.9, Multiband-SENSE factor = 2, flip angle of 90°, FOV=224x224 mm^2^, 66 slices for an acquisition time of 9 min 19s. The data comprised of 100 unique directions uniformly spread over three shells at b = 300 mm^2^/s (n=8 directions), b = 1000 mm^2^/s (n=32 directions), b = 2000 mm^2^/s (n=60 directions), with non-diffusion-weighted images b = 0 mm^2^/s (n=7), for a total of 107 total diffusion volume (Caruyer et al., 2013). To correct EPI distortions, a reversed phase-encoded b = 0 image was acquired right after the DWI acquisition, with the same geometry (Andersson et al., 2003).

(c) Inhomogeneous MT images were acquired using a 3D segmented-EPI gradient-echo sequence with different MT preparation pulses with first TE/TR = 3.6/112 ms, 2 x 2 mm resolution, flip angle of 15°, FOV=224x224 mm, 65 slices of 2 mm of thickness and 3 echoes with echo spacing 6.0 ms for an acquisition time of 6 min 04s. Inhomogeneous MT uses a magnetization preparation (10 Hann pulses of 0.9 ms duration with 1.5 ms interval at a frequency offset of +/- 7000 Hz) (Varma et al., 2015). Two additional reference images were acquired for each echo without MT preparation, with the same parameters as the MT sequence, a second with a higher flip angle (30°), and a shorter TR (20 ms) for quantification purposes.

(d) 3D FLAIR images were obtained with the following parameters: TR/TE/inversion time (TI)= 4800/340/1650 ms, flip angle of 90°, FOV=224x224 mm^2^, data was acquired at 1.12x1.12mm^3^ for 163 slices of 1.12 mm of thickness and reconstructed at voxel size 0.74x0.74x0.56 mm^3^, as in the clinical routine, for an acquisition time of 4 min 33s.

| **Sequences**  **Parameters** | **T1** | **FLAIR** | **DWI** | **Reverse B0** | **ihMT** | **T1 ihMT** |
| --- | --- | --- | --- | --- | --- | --- |
| **Direction** | Axial | Sagittal | Axial | Axial | Axial | Axial |
| **Technique – Fast imaging method** | FFE – TFE | IR – TSE | SE – EPI | SE – EPI | FFE – EPI | FFE – EPI |
| **Durée** | 4 min 20s | 4 min 33s | 9 min 20s | 14 s | 6 min 04s | 13 s |
| **TR (ms)** | 7.9 | 4800 | 4800 | 4800 | 112 | 20 |
| **TE (ms)** | 3.5 | 340 | 92 | 92 | 3.6 (Δ=6) | 3.6 (Δ=6) |
| **TI (ms)** |  | 1650 |  |  |  |  |
| **Flip Angle (degree)** | 8 | 90 | 90 | 17 | 15 | 30 |
| **FOV (mm)** | 224x224 | 250x250 | 224 x 224 | 228x180 | 224x224 | 224x224 |
| **Slice** | 150 | 326 | 132 | 162 | 65 | 65 |
| **Voxel size (mm)** | 1x1x1 | 0.74x0.74  x0.56 (reconstructed) | 2x2x2 | 2x2x2 | 2x2x2 | 2x2x2 |
| **n b0, b-value (n directions)** |  |  | 7, 300 (8), 1000 (32),2000 (60) |  |  |  |
| **Pulse** |  |  |  |  | 10 Hann pulses of 0.9 ms duration with 1.5 ms interval |  |
| **Frequency offset of +/-** |  |  |  |  | 7000 Hz |  |
| **Echoes – Echo spacing** |  |  |  |  | 3 – 6 ms |  |

**Table 1**. Acquisition parameters for each sequence.

1. **The time interval for the different study designs**

|  | **Healthy controls dataset** | | | | **RR-MS dataset** | | |
| --- | --- | --- | --- | --- | --- | --- | --- |
|  | | Mean | SD | Mean | | SD |  |
| **Number of days between MRI follow-up** | | 30.4 | 4.2 | 34.8 | | 5.0 |  |
|  | |  |  |  | |  |  |
| **Number of days between baseline and last follow-up** | | | | | | | |
| Designs | | Mean | SD | Mean | | SD | |
| D_012_ | | 60.9 | 5.1 | 69.7 | | 5.8 | |
| D_013_, D_023_ | | 92.0 | 4.6 | 105.7 | | 6.8 | |
| D_014_, D_024_, D_034_ | | 121.6 | 4.9 | 139.4 | | 5.9 | |

**Table 2.** The average number of days between each MRI follow-up and between the baseline and last follow-up according to the different designs.

1. **Lesion segmentation**

Lesion segmentation was performed on the baseline T1-weighted and FLAIR images (acquisition 1) by ME and EL. First, baseline FLAIR images were linearly co-registered and resampled to baseline T1-weighted image space (1 mm3 isotropic voxels) for each subject using Anatomical Normalization Tools (ANTs, Avants et al., 2008). Next, lesions were manually segmented by using interactive open-source software ITK-Snap (version 3.6; [www.itksnap.org](http://www.itksnap.org), Yushkevich et al., 2006). ITK-Snap allows visualization and segmentation in the three planes (axial, sagittal and coronal) and switching between each image contrast. For the segmentation, lesions were identified in FLAIR images and border delimitation was done in T1-weighted images with a back-and-forth between each contrast if needed. For each section, the segmentation was firstly performed on the axial plane and verified in the sagittal and coronal planes. Finally, sections above and below were also examined to limit ambiguities and evaluate the shape of the final lesion in 3D. For the follow-up (acquisitions 2 to 5), FLAIR and T1-weighted follow-up images were firstly linearly co-registered to the baseline images (acquisition 1). For each acquisition, the previous acquisition segmentation was used as a reference and modified successively to add all new lesions observed in the current acquisition and not present at baseline MRI. The segmentation lesion definition follows the same process as the baseline. A new lesion was defined as the presence of at least one lesion with a diameter of 2 mm (2 voxels) or a significantly enlarged lesion on follow-up images (Thompson et al., 2018). To obtain the segmentation of the lesions in the native space, the inverse transformation was applied to the segmentation of each follow-up.

**Results supplementary.**

1. **Healthy controls dataset supplementary**
   1. **Correlation between MRI change over time from study designs for the Healthy controls’ dataset**

A statistical description of the changes over time of the MRI measurements for both datasets is reported in Supplementary Table 3.

We also assessed the similarity between the reference design and the tested designs. The Pearson correlation coefficient was used as a measure of similarity between the changes in MRI parameters from the tested designs and those obtained from the reference design for each bundle and MRI measure. A moderate (D012 [r=0.68]) to strong (D034 [r=0.89]) similarity is found for changes in MRI parameters with the tested designs compared to the reference design, regardless of the bundles and MRI measures. The similarity is equivalent across bundles. However, a variable level of similarity is observed for the MRI measures, with ISOVF showing the lowest level of similarity (r=0.68), while AFD total shows the highest level on average (r=0.93).

| **Pearson correlation coefficient between change over time from reference design and tested designs – Healthy controls’ dataset** | | | | | | | |
| --- | --- | --- | --- | --- | --- | --- | --- |
|  | **Same study duration** | | | **Shorter study duration** | | |  |
|  | D_014_ | D_024_ | D_034_ | D_012_ | D_013_ | D_023_ | Average |
| **Reference design** | 0.836 | 0.834 | 0.888 | 0.673 | 0.772 | 0.765 | 0.795 |
|  |  |  |  |  |  |  |  |
| **Bundles** |  |  |  |  |  |  |  |
| CC3 | 0.789 | 0.891 | 0.944 | 0.734 | 0.726 | 0.777 | 0.810 |
| CG | 0.917 | 0.938 | 0.923 | 0.614 | 0.843 | 0.804 | 0.840 |
| CST | 0.876 | 0.849 | 0.904 | 0.688 | 0.745 | 0.755 | 0.803 |
| OR | 0.774 | 0.674 | 0.822 | 0.671 | 0.804 | 0.790 | 0.756 |
| SLF2 | 0.823 | 0.818 | 0.847 | 0.659 | 0.742 | 0.698 | 0.765 |
|  |  |  |  |  |  |  |  |
| **MRI measures** |  |  |  |  |  |  |  |
| MD | 0.756 | 0.888 | 0.870 | 0.565 | 0.796 | 0.683 | 0.760 |
| RD | 0.812 | 0.719 | 0.799 | 0.812 | 0.713 | 0.685 | 0.757 |
| ihMTdR1sat | 0.952 | 0.807 | 0.888 | 0.752 | 0.797 | 0.687 | 0.814 |
| MTR | 0.807 | 0.758 | 0.914 | 0.817 | 0.726 | 0.804 | 0.804 |
| AFD total | 0.951 | 0.982 | 0.956 | 0.738 | 0.972 | 0.956 | 0.926 |
| ICVF | 0.845 | 0.886 | 0.986 | 0.487 | 0.634 | 0.935 | 0.795 |
| ISOVF | 0.753 | 0.720 | 0.684 | 0.474 | 0.757 | 0.656 | 0.674 |
| FW | 0.775 | 0.848 | 0.965 | 0.672 | 0.823 | 0.731 | 0.802 |

**Table 4.** Pearson correlation coefficient between reference design and each tested design for each bundle and MRI measures for the Healthy dataset.

- 1. **Sample sizes estimation for the Healthy controls’ dataset**

Here, we provide the mean number of sample sizes required for each design and MRI measure, regardless of the bundles using a power of 0.8 and α of 0.05 for ANOVA analyses in the healthy controls’ dataset.

| Sample size estimation for Healthy controls’ dataset | | | | | | | | |
| --- | --- | --- | --- | --- | --- | --- | --- | --- |
| Designs  MRI measure | **Same study duration** | | |  | **Shorter study duration** | | | **Mean by** |
|  | D_014_ | D_024_ | D_034_ | D_R_ | D_012_ | D_013_ | D_023_ | **measure** |
| MD | 19.2 | 18.4 | 18.2 | 14.4 | 23.0 | 21.2 | 21.4 | 19.40 |
| RD | 16.8 | 14.4 | 15.8 | 11.8 | 18.6 | 17.6 | 16.2 | 15.89 |
| ihMTdR1sat | 54.8 | 48.0 | 52.0 | 35.4 | 48.6 | 49.4 | 41.2 | 47.06 |
| MTR | 47.2 | 53.8 | 46.8 | 33.0 | 55.6 | 50.6 | 52.4 | 48.49 |
| AFD total | 31.6 | 32.4 | 18.4 | 24.2 | 38.8 | 35.6 | 29.6 | 30.09 |
| ICvf | 15.6 | 15.8 | 16.4 | 12.2 | 18.8 | 16.2 | 17.2 | 16.03 |
| ISOvf | 26.2 | 25.4 | 31.0 | 20.2 | 22.0 | 31.4 | 30.2 | 26.63 |
| FW | 19.0 | 21.4 | 19.4 | 15.2 | 22.2 | 21.4 | 22.0 | 20.09 |
| Mean by design | 28.80 | 28.70 | 27.25 | 20.8 | 30.95 | 30.43 | 28.78 |  |

**Table 5**. Sample size corresponds to the reference design and each tested design, and metrics for the Healthy controls’ dataset. The sample size was estimated using G*Power 3.1 to achieve a statistical power of 0.8 and significance of alpha=0.05 for Group (1) x Time (5 or 3) within groups ANOVA with a small effect size (f = 0.2). The number represents the average sample size across bundles. Red colors indicate the highest number of subjects and blue colors the lowest number.

1. **RR-MS dataset supplementary**
   1. **Correlation between MRI change over time from study designs for the RR-MS dataset**

For the RR-MS dataset, a moderate (D012 [r=0.62]) to strong (D034 [r=0.87]) similarity is found for changes in MRI parameters with the tested designs compared to the reference design, regardless of the bundles and MRI measures. An equivalent similarity is observed across bundles. However, a variable level of similarity is observed for the MRI measures, with RD showing the lowest level of similarity (r=0.57), while ICVF shows the highest level on average (r=0.89).

| **Pearson correlation coefficient between change over time from reference design and tested designs – RR-MS dataset** | | | | | | | |
| --- | --- | --- | --- | --- | --- | --- | --- |
|  | **Same study duration** | | | **Shorter study duration** | | |  |
|  | D_014_ | D_024_ | D_034_ | D_012_ | D_013_ | D_023_ | **Average** |
| **Reference design** | 0.780 | 0.734 | 0.865 | 0.616 | 0.809 | 0.849 | 0.775 |
|  |  |  |  |  |  |  |  |
| **Bundles** | | | | | | | |
| CC 3 | 0.821 | 0.540 | 0.888 | 0.633 | 0.783 | 0.829 | 0.749 |
| CG | 0.837 | 0.810 | 0.870 | 0.817 | 0.885 | 0.899 | 0.853 |
| CST | 0.800 | 0.831 | 0.762 | 0.713 | 0.758 | 0.752 | 0.769 |
| OR | 0.644 | 0.714 | 0.912 | 0.258 | 0.870 | 0.923 | 0.720 |
| SLF 2 | 0.797 | 0.777 | 0.891 | 0.659 | 0.749 | 0.840 | 0.785 |
|  |  |  |  |  |  |  |  |
| **MRI measures** | | | | | | | |
| MD | 0.766 | 0.652 | 0.775 | 0.774 | 0.869 | 0.799 | 0.772 |
| RD | 0.341 | 0.198 | 0.894 | 0.153 | 0.875 | 0.951 | 0.569 |
| ihMTdR1sat | 0.768 | 0.829 | 0.951 | 0.550 | 0.614 | 0.474 | 0.698 |
| MTR | 0.942 | 0.884 | 0.821 | 0.750 | 0.806 | 0.824 | 0.838 |
| AFD total | 0.811 | 0.869 | 0.929 | 0.643 | 0.876 | 0.940 | 0.845 |
| ICVF | 0.926 | 0.834 | 0.967 | 0.685 | 0.933 | 0.953 | 0.883 |
| ISOVF | 0.888 | 0.776 | 0.846 | 0.746 | 0.765 | 0.960 | 0.830 |
| FW | 0.881 | 0.695 | 0.856 | 0.690 | 0.735 | 0.961 | 0.803 |

**Table 6.** Pearson correlation coefficient between reference design and each tested design for each bundle and MRI measures for the RR-MS dataset.

- 1. **Sample sizes estimation for the RR-MS dataset**

The number of subjects required varies according to the design, i.e., tested designs over the same study duration require more subjects than those over different study durations. The number of subjects required also varies according to the bundle studied and the MRI measures used. CST requires more subjects than other bundles. Similarly, the ihMTsat, MTR, AFD Total, ISOvf and ICvf measures require more subjects than the MD, RD and FW measures (Figure 6).

| Sample size estimation for RR-MS dataset | | | | | | | | |
| --- | --- | --- | --- | --- | --- | --- | --- | --- |
| Designs  MRI measure | **Same study duration** | | |  | **Shorter study duration** | | | **Mean** |
|  | D_014_ | D_024_ | D_034_ | D_R_ | D_012_ | D_013_ | D_023_ | **by measure** |
| MD | 61.6 | 62.4 | 59.0 | 39.8 | 36.2 | 48.6 | 49.4 | 51.0 |
| RD | 63.4 | 63.4 | 62.2 | 46.2 | 33.6 | 47.2 | 49.8 | 52.26 |
| ihMTdR1sat | 63.0 | 76.6 | 75.2 | 50.0 | 63.6 | 65.2 | 73.6 | 66.74 |
| MTR | 84.0 | 83.4 | 88.8 | 47.4 | 37.8 | 41.8 | 41.2 | 60.63 |
| AFD total | 104.4 | 110.2 | 107.4 | 64.6 | 73.8 | 52.6 | 81.8 | 84.97 |
| ICvf | 77.2 | 77.8 | 76.2 | 45.8 | 44.2 | 38.8 | 43.4 | 57.63 |
| ISOvf | 89.6 | 87.2 | 82.8 | 71.2 | 38.0 | 46.8 | 47.4 | 66.14 |
| FW | 53.4 | 57.8 | 48.8 | 31.6 | 28.6 | 29.8 | 29.8 | 39.97 |
| Mean by design | 74.58 | 77.35 | 75.05 | 49.58 | 44.48 | 46.35 | 52.05 |  |

**Table 7.** Sample size corresponds to the reference design and each tested design, and metrics for the MS dataset. The sample size was estimated using G*Power 3.1 to achieve a statistical power of 0.8 and significance of alpha=0.05 for Group (1) x Time (5 or 3) within groups ANOVA with a small effect size (f = 0.2). The number represents the average sample size across bundles. Red colors indicate the highest number of subjects and blue colors the lowest number.

- 1. **Clinical outcome associations for the RR-MS dataset**

Note that new associations (i.e., not found for the reference design) are found for all tested designs except for the shortest tested design D012. The number of new associations is reported in Table 7 and Figure 1.

| **Number of new associations for tested designs – RR-MS dataset** | | | | | | | |
| --- | --- | --- | --- | --- | --- | --- | --- |
|  | **Same study duration** | | | **Shorter study duration** | |  |  |
|  |  |  |  |  |  |  |  |
| **Designs (n)** | D_014_ | D_024_ | D_034_ | D_013_ | D_023_ | Total | Mean |
|  | 34 | 29 | 49 | 60 | 61 | 121 | 60.5 |
|  |  |  |  |  |  |  |  |
| **MRI models (n)** |  |  |  |  |  |  |  |
| DTI | 7 | 10 | 8 | 9 | 5 | 39 | 7.8 |
| NODDI | 12 | 13 | 5 | 4 | 8 | 42 | 8.4 |
| HARDI | 13 | 8 | 3 | 1 | 8 | 33 | 6.6 |
| MTI | 15 | 17 | 11 | 8 | 12 | 63 | 12.6 |
|  |  |  |  |  |  |  |  |
| **Bundles (n)** |  |  |  |  |  |  |  |
| AF | 1 | 1 | 3 | 3 | 2 | 5 | 2.5 |
| CC 2a | 0 | 0 | 1 | 5 | 5 | 10 | 5.0 |
| CC 2b | 1 | 1 | 0 | 1 | 2 | 3 | 1.5 |
| CC 3 | 0 | 0 | 2 | 5 | 3 | 8 | 4.0 |
| CC 4 | 2 | 4 | 3 | 4 | 1 | 5 | 2.5 |
| CC 5 | 4 | 5 | 8 | 7 | 10 | 17 | 8.5 |
| CC 6 | 4 | 0 | 1 | 3 | 5 | 8 | 4.0 |
| CC 7 | 2 | 2 | 2 | 4 | 6 | 10 | 5.0 |
| CG | 0 | 2 | 3 | 2 | 1 | 3 | 1.5 |
| CST | 6 | 2 | 8 | 5 | 5 | 10 | 5.0 |
| IFOF | 3 | 1 | 3 | 5 | 5 | 10 | 5.0 |
| ILF | 4 | 3 | 7 | 5 | 6 | 11 | 5.5 |
| OR | 4 | 4 | 2 | 1 | 2 | 3 | 1.5 |
| SLF 1 | 2 | 2 | 2 | 6 | 5 | 11 | 5.5 |
| SLF 2 | 0 | 0 | 0 | 5 | 5 | 10 | 5.0 |
| SLF 3 | 0 | 0 | 2 | 7 | 5 | 12 | 6.0 |
| UF | 8 | 6 | 1 | 5 | 1 | 6 | 3.0 |
| n: number of new or preserved association | | | |  |  |  |  |

**Table 8.** The number of new associations corresponds to tested designs for each MRI model and bundle in RR-MS dataset.

The shorter study duration designs D013 and D023 have the highest frequency of new associations (number of new associations ≥ 60) compared to the others (n < 50). The CC 5, CST, ILF and UF bundles (n > 4 on average), and the MTI-derived measures (n ~ 12 on average) also have higher frequencies compared to other bundles (n ≤ 3 on average) and MRI designs (n ≤ 8 on average).

**Figure 1.** The number of new associations in RR-MS dataset. A) Bars represent the number of new associations for each design and B) for each MRI model. C) Stacked bars represent the cumulative number of new associations for each bundle and each design. The color represents the design.

**Supplementary References**

Andersson, J.L.R., Skare, S., Ashburner, J., 2003. How to correct susceptibility distortions in spin-echo echo-planar images: application to diffusion tensor imaging. NeuroImage 20, 870–888. https://doi.org/10.1016/S1053-8119(03)00336-7

Avants, B.B., Epstein, C.L., Grossman, M., Gee, J.C., 2008. Symmetric diffeomorphic image registration with cross-correlation: evaluating automated labeling of elderly and neurodegenerative brain. Med Image Anal 12, 26–41. https://doi.org/10.1016/j.media.2007.06.004

Caruyer, E., Lenglet, C., Sapiro, G., Deriche, R., 2013. Design of multishell sampling schemes with uniform coverage in diffusion MRI. Magnetic Resonance in Medicine 69, 1534. https://doi.org/10.1002/mrm.24736

Kieseier, B.C., Pozzilli, C., 2012. Assessing walking disability in multiple sclerosis. Mult Scler 18, 914–924. https://doi.org/10.1177/1352458512444498

Polman, C.H., Rudick, R.A., 2010. The Multiple Sclerosis Functional Composite: A clinically meaningful measure of disability. Neurology 74, S8–S15. https://doi.org/10.1212/WNL.0b013e3181dbb571

Rosti-Otajärvi, E., Hämäläinen, P., Koivisto, K., Hokkanen, L., 2008. The reliability of the MSFC and its components. Acta Neurol Scand 117, 421–427. https://doi.org/10.1111/j.1600-0404.2007.00972.x

Rudick, R., Antel, J., Confavreux, C., Cutter, G., Ellison, G., Fischer, J., Lublin, F., Miller, A., Petkau, J., Rao, S., Reingold, S., Syndulko, K., Thompson, A., Wallenberg, J., Weinshenker, B., Willoughby, E., 1997. Recommendations from the National Multiple Sclerosis Society Clinical Outcomes Assessment Task Force. Ann Neurol 42, 379–382. https://doi.org/10.1002/ana.410420318

Thompson, A.J., Baranzini, S.E., Geurts, J., Hemmer, B., Ciccarelli, O., 2018. Multiple sclerosis. The Lancet 391, 1622–1636. https://doi.org/10.1016/S0140-6736(18)30481-1

Varma, G., Girard, O.M., Prevost, V.H., Grant, A.K., Duhamel, G., Alsop, D.C., 2015. Interpretation of magnetization transfer from inhomogeneously broadened lines (ihMT) in tissues as a dipolar order effect within motion restricted molecules. Journal of Magnetic Resonance 260, 67–76. https://doi.org/10.1016/j.jmr.2015.08.024

Yushkevich, P.A., Piven, J., Hazlett, H.C., Smith, R.G., Ho, S., Gee, J.C., Gerig, G., 2006. User-guided 3D active contour segmentation of anatomical structures: Significantly improved efficiency and reliability. NeuroImage 31, 1116–1128. https://doi.org/10.1016/j.neuroimage.2006.01.015
